# Supplementary material for: Presynaptic NMDARs cooperate with local spikes toward GABA release from the reciprocal olfactory bulb granule cell spine
Source: eLife. 2020 Nov 30;9:e63737. doi: 10.7554/eLife.63737 (PMC7704106; doi:10.7554/eLife.63737)
Supplement: Transparent reporting form [file elife-63737-transrepform.docx]

***eLife’s* transparent reporting form**

We encourage authors to provide detailed information *within their submission* to facilitate the interpretation and replication of experiments. Authors can upload supporting documentation to indicate the use of appropriate reporting guidelines for health-related research (see [EQUATOR Network](http://www.equator-network.org/%20)), life science research (see the [BioSharing Information Resource](https://biosharing.org/)), or the [ARRIVE guidelines](http://www.plosbiology.org/article/info:doi/10.1371/journal.pbio.1000412) for reporting work involving animal research. Where applicable, authors should refer to any relevant reporting standards documents in this form.

If you have any questions, please consult our Journal Policies and/or contact us: [editorial@elifesciences.org](mailto:editorial@elifesciences.org).

**Sample-size estimation**

- You should state whether an appropriate sample size was computed when the study was being designed
- You should state the statistical method of sample size computation and any required assumptions
- If no explicit power analysis was used, you should describe how you decided what sample (replicate) size (number) to use

Please outline where this information can be found within the submission (e.g., sections or figure legends), or explain why this information doesn’t apply to your submission:

Uncaging data: No explicit power analysis was used, since the type of experiment (uncaging of glutamate and recording of reciprocal IPSC) was conducted for the first time.

For control type experiments (e.g. stability of urIPSC recordings, GABAA receptor pharmacology), we attempted to obtain a minimum of N = 7 experiments which can yield significance levels < 0.01 in the non-parametric Wilcoxon test (which we have been using routinely since many years, if the type of experiment was novel and normal distributions cannot be assumed). This is stated in the Statistics section of the Methods part. For the experiments that were at the core of our interest (Nav channel blockade, NMDA receptor blockade) we attempted to obtain at least N = 10 experiments. I also wish to point out that these experiments are very difficult to conduct.

Ultrastructure data: We analysed as many segments as were detectable in the pre-existing sections (selected according to the criterion that synaptic profiles – either symmetric or asymmetric or both - between mitral cell dendrites and granule cell spines were present, as stated in the Methods).

**Replicates**

- You should report how often each experiment was performed
- You should include a definition of biological versus technical replication
- The data obtained should be provided and sufficient information should be provided to indicate the number of independent biological and/or technical replicates
- If you encountered any outliers, you should describe how these were handled
- Criteria for exclusion/inclusion of data should be clearly stated
- High-throughput sequence data should be uploaded before submission, with a private link for reviewers provided (these are available from both GEO and ArrayExpress)

Please outline where this information can be found within the submission (e.g., sections or figure legends), or explain why this information doesn’t apply to your submission:

All of our data pertain to biological replicates. This is stated in the Methods subsection on statistical analysis. N numbers are always denoted with the items they refer to (cells, segments, traces… ).

The numbers of replicates are always stated both in the respective Figure legends and along with the measurement outcomes in the Results text.

The data analysis is described in detail in the Methods section. Outliers were never excluded, again since the uncaging experiments are novel; also, we know from our previous investigations about special sources of variability, e.g. some spines might not have Nav channels at all etc. (see e.g Fig. 1E left, Fig. 2D left, Fig S4 B). Individual data points are shown in all the cumulative data displays (Fig 1- 4, 6).

**Statistical reporting**

- Statistical analysis methods should be described and justified
- Raw data should be presented in figures whenever informative to do so (typically when N per group is less than 10)
- For each experiment, you should identify the statistical tests used, exact values of N, definitions of center, methods of multiple test correction, and dispersion and precision measures (e.g., mean, median, SD, SEM, confidence intervals; and, for the major substantive results, a measure of effect size (e.g., Pearson's r, Cohen's d)
- Report exact p-values wherever possible alongside the summary statistics and 95% confidence intervals. These should be reported for all key questions and not only when the p-value is less than 0.05.

Please outline where this information can be found within the submission (e.g., sections or figure legends), or explain why this information doesn’t apply to your submission:

The used statistical tests and the rationale for choosing them are described in the Statistics part of the Methods section.

Individual data points/raw data of physiology experiments are shown in all the cumulative data displays (Fig. 1- 4, Fig S1, S2, S3, S4). For the histograms in Fig 1, the raw data are given in the additional source data. For the ultrastructural data, the raw density data are evident from the cumulative probability plots (Fig 6 B,C). Exact p-values are always indicated.

For the simulations (Fig. 5), we conducted a robustness analysis for the most important model parameters with regard to the amount of overlap between NMDAR and HVA Ca2+ currents (Fig. 5C).

(For large datasets, or papers with a very large number of statistical tests, you may upload a single table file with tests, Ns, etc., with reference to sections in the manuscript.)

**Group allocation**

- Indicate how samples were allocated into experimental groups (in the case of clinical studies, please specify allocation to treatment method); if randomization was used, please also state if restricted randomization was applied
- Indicate if masking was used during group allocation, data collection and/or data analysis

Please outline where this information can be found within the submission (e.g., sections or figure legends), or explain why this information doesn’t apply to your submission:

Randomization did not play a role in our study. No masking was used for the physiology data, since they were analysed by the experimenter. The student who analysed the ultrastructural data under the guidance of Dr Sassoe-Pognetto was not blind with regard to the purpose of the investigation. However, all crucial analyses were double-checked by a second, more senior person. For the simulations, we used the pre-existing model from Aghvami et al. 2019; no tweaking of parameters happened (this is also stated explicitly in the Results section).

**Additional data files (“source data”)**

- We encourage you to upload relevant additional data files, such as numerical data that are represented as a graph in a figure, or as a summary table
- Where provided, these should be in the most useful format, and they can be uploaded as “Source data” files linked to a main figure or table
- Include model definition files including the full list of parameters used
- Include code used for data analysis (e.g., R, MatLab)
- Avoid stating that data files are “available upon request”

Please indicate the figures or tables for which source data files have been provided:

Fig 1,2,3,4,5,6; Fig. S1, S2, S3, S4

Data analysis is described in a rather detailed way in the methods section.

As to the simulations, they are based on an already published model (ModelDB entry 244687) with the very same parameters. A new modelDB entry will be made upon acceptance that also contains the code for the robustness testing (Fig. 5C). All the relevant source code is included in the source data files for Fig 5, as well as the results of the robustness testing.
